# The Role of Long Non-Coding RNAs in Trophoblast Regulation in Preeclampsia and Intrauterine Growth Restriction

**DOI:** 10.3390/genes12070970

**Published:** 2021-06-25

**Authors:** Lara J. Monteiro, Reyna Peñailillo, Mario Sánchez, Stephanie Acuña-Gallardo, Max Mönckeberg, Judith Ong, Mahesh Choolani, Sebastián E. Illanes, Gino Nardocci

**Affiliations:** 1 Faculty of Medicine, Universidad de los Andes, Santiago 7620001, Chile; lmonteiro@uandes.cl (L.J.M.); stephanie.acuna.gallardo@gmail.com (S.A.-G.); mmonckebergz@uandes.cl (M.M.); sillanes@uandes.cl (S.E.I.); 2 Laboratory of Reproductive Biology, Center for Biomedical Research and Innovation (CIIB), Universidad de los Andes, Santiago 7620001, Chile; rpenailillo@uandes.cl; 3 Molecular Biology and Bioinformatics Lab, Program in Molecular Biology and Bioinformatics, Center for Biomedical Research and Innovation (CIIB), Universidad de los Andes, Santiago 7620001, Chile; mario.sanchez.rubio@gmail.com; 4 Yong Loo Lin School of Medicine, National University of Singapore, Singapore 119228, Singapore; judith_ong@nuhs.edu.sg (J.O.); obgmac@nus.edu.sg (M.C.); 5 Department of Obstetrics and Gynaecology, National University Hospital, Singapore 119228, Singapore

**Keywords:** long non-coding RNAs, preeclampsia, intrauterine growth restriction

## Abstract

Preeclampsia (PE) and Intrauterine Growth Restriction (IUGR) are two pregnancy-specific placental disorders with high maternal, fetal, and neonatal morbidity and mortality rates worldwide. The identification biomarkers involved in the dysregulation of PE and IUGR are fundamental for developing new strategies for early detection and management of these pregnancy pathologies. Several studies have demonstrated the importance of long non-coding RNAs (lncRNAs) as essential regulators of many biological processes in cells and tissues, and the placenta is not an exception. In this review, we summarize the importance of lncRNAs in the regulation of trophoblasts during the development of PE and IUGR, and other placental disorders.

## 1. Introduction

The placenta constitutes the interface for fetal-maternal intercommunication and has a crucial role in regulating fetal resources. Thus, this organ is vital for fetal well-being; is responsible for providing protection and delivering oxygen and nutrients to the growing fetus [1,2,3]. Defects that compromise placenta function are related to a wide range of pregnancy disorders that can alter fetal growth, produce late pregnancy disorders, and even lifelong metabolic reprogramming both in the mother and the offspring [4,5].

Preeclampsia (PE) and Intrauterine Growth Restriction (IUGR) are two pregnancy-specific multisystem disorders that are significant contributors to maternal, fetal, and neonatal morbidity and mortality rates worldwide. PE is a heterogeneous and multisystemic pregnancy-associated disorder present in up to 5% of pregnancies worldwide and is clinically characterized as de novo development of hypertension (>140/90 mmHg) accompanied with proteinuria (>0.3 g in 24 h urine) at 20 weeks of gestation [6,7]. This pathology is associated with an increased risk of maternal and fetal morbidity and mortality. Approximately two-thirds of preeclamptic pregnancies are complicated with fetal growth restriction and increased susceptibility to chronic diseases later in life [8]. On the other hand, IUGR is defined as a failure of the fetus to reach a predetermined growth potential. It affects 10–15% of all pregnancies, and it is associated with increased risk for immediate (metabolic and hematological disturbances, disrupted thermoregulation, respiratory distress, necrotizing enterocolitis, retinopathy of prematurity) and long-term (cardiovascular diseases, dyslipidemia, type 2 diabetes mellitus, obesity, and metabolic syndrome) consequences for the offspring [9,10]. Furthermore, PE and IUGR can quickly progress to life-threatening disorders [11,12,13,14,15].

Identifying molecules or genes dysregulated in PE and IUGR is fundamental for understanding their physiopathology and can contribute decisively to developing early detection and management strategies. In recent years, long non-coding RNAs (lncRNAs) have gained importance as regulators of gene expression in several biological processes in humans and other organisms. These RNA molecules are currently a hot topic in biological research due to their essential regulatory mechanisms in mammalian homeostasis and pathogenesis [16,17,18]. Regulation of lncRNAs has been linked to essential roles in many fundamental biological processes, including development, metabolism, immunity, cancer, and epigenetic control [19,20,21,22,23,24]. The placenta is not excluded by the control of these non-coding RNAs, although relatively few studies have focused on the role of lncRNAs during placenta development and the onset of placental-associated disorders.

Due to the essential role of lncRNAs in gene regulation, these molecules have been highlighted as potential biomarkers for the diagnosis and prognosis of different pathologies such as various cancer types, pregnancy pathologies, cardiovascular diseases, and neurodegenerative disorders, among others [25,26,27,28,29,30,31,32]. A better understanding of the role of lncRNAs in normal and abnormal placental development can be of the utmost importance to explain the processes underlying the pathogenesis of some pregnancy complications. Therefore, identifying lncRNAs of placental origin in conditions that endanger maternal and fetal health can shed light on developing new predictive and therapeutic strategies against placental disorders such as PE or IUGR.

## 2. LncRNAs: Definition and Classification

The central dogma of molecular biology indicates that DNA is transcribed into a messenger RNA, which serves as a template for protein synthesis [33]. In this dogma, proteins have been the main protagonists of cellular functions, while RNA functions have only been described as an intermediary between DNA sequences and the proteins they encode. However, less than 2% of the genomic sequences encode for proteins, while almost 90% of the entire genome is actively transcribed [34,35,36]. In recent years, several studies have revealed a series of RNAs that have no potential to encode proteins but are involved in different cellular processes, offering new perspectives on the centrality of RNA in the dogma of molecular biology [19,33,37,38]. These transcripts are known as non-coding RNAs (ncRNAs), and many of them have been shown to play a crucial role in the development and epigenetic regulation of gene expression. Interestingly, identifying specific profiles of ncRNAs has also been linked to different diseases [19,39,40].

The non-coding transcripts are classified into two broad groups according to their size: ncRNAs with 200 or less nucleotides (nt), namely, small RNAs; and non-coding RNAs greater than 200 nucleotides also know as long non-coding RNAs (lncRNAs). Within the former group we can highlight microRNAs (miRNAs), RNAs that interact with PIWI (piRNA), and small RNAs of endogenous interference (siRNA). Their primary function is associated with gene transcriptional silencing [41,42,43,44]. On the other hand, lncRNAs, which represent the most diverse and extensive group of ncRNAs, are involved in different biological processes, such as imprinting, cell cycle control, nucleus-cytoplasm transport, nuclear architecture, transcriptional and post-transcriptional regulation, and epigenetic regulation, among others [19,20,33,37,38,45,46,47,48,49].

Most lncRNAs are structurally similar to messenger RNAs, but they lack an open reading frame. Many of these lncRNAs are transcribed by the RNA polymerase II, capped at the 5′ end, and polyadenylated at the 3′ end. They can interact directly with DNA or RNAs through base pairing, just as they can interact with proteins through their secondary and tertiary structures. They are located both in the nucleus and in the cytoplasm, and they are expressed at very low space-time levels during the development of cells, tissues, and organs, which suggests they have functions that can affect any of those processes [19,39,40,50,51,52,53]. Moreover, lncRNAs can regulate gene expression through multiple molecular mechanisms. These mechanisms include: (i) Acting as molecular “decoys” binding to transcription factors mimicking their target site and preventing binding to their targets [54], (ii) acting as molecular “guides,” to allow the correct localization of a specific protein complex (i.e., recruiting chromatin-modifying enzymes to target genes) [55], (iii) work as molecular “scaffolds” by recruiting epigenetic modifiers (i.e., ribonucleoprotein complex), (iv) act as miRNAs “sponges,” preventing their union to their target site [56,57], or (v) can act as enhancer-derived RNAs (eRNAs), which are transcribed at the loci of enhancers and are involved in gene activation and in the formation of higher-order chromatin structures [58,59,60]. Thus, depending on their nature, lncRNAs can be involved in both gene silencing and transcriptional activation [20,33,36,48,49,61].

**Figure 1:**
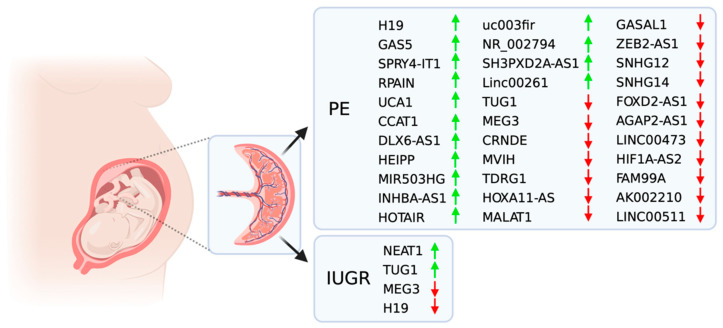
Overview of lncRNA associated with Preeclampsia (PE) and Intrauterine Growth Restriction (IUGR). The green and red arrows indicate upregulated or downregulated lncRNAs, respectively, in these pregnancy pathologies. Created with BioRender.com.

Specific lncRNAs may be involved in the onset of different pathologies [17,19,20,23,62], and pregnancy-related complications are an example of this phenomenon. Placental-related disorders such as PE and IUGR result from defective early placental development, which later impacts placental function and, thereby, the mother and fetus’s health [4,7,8,9,13]. LncRNAs can play a crucial role in a healthy and functional placenta and may present aberrantly over- or under-expressed pathologies with inadequate placentation (Figure 1). Therefore, due to their highly tissue- and disease-specific expression, it is crucial to study lncRNAs in order to understand these diseases and to be able to develop new therapeutic targets and preventive strategies. Thus far, using these ncRNAs remains relatively unexplored in novel drug targets or diagnostic biomarkers in pregnancy-related pathologies.

## 3. Etiopathogenesis of Placental Related Diseases

The establishment of a functional placenta is essential for normal fetal development and the maintenance of pregnancy. When the blastocyst approaches the epithelia of the endometrium during the window of implantation, the outer epithelial monolayer of cells, the trophectoderm, differentiates into two regions: (i) Syncytiotrophoblast, a multi-nucleic cell outer layer that penetrates the uterine epithelium, allowing the embryo to embed itself within the endometrium; and (ii) mononuclear cytotrophoblast cells that proliferate to form cell columns that invade into maternal tissues [63,64,65]. From the tips of these anchoring villi structures, extravillous cytotrophoblast (EVT) cells emerge. These cells are highly migratory, proliferative, and invasive [66]. Trophoblast invasion of the endometrium involves attachment of these cells to the extracellular matrix and then degradation of this matrix with the subsequent migration [66]. There are two populations of EVT: Interstitial cytotrophoblasts, which invade the entire endometrium and the superficial myometrium, and endovascular cytotrophoblasts, which invade the lumen of the spiral arteries and transform them from high-resistance vessels into large, dilated vessels with increased blood flow at a much-reduced pressure [65,67]. When trophoblast cells fail to effectively invade the maternal decidua or sufficiently remodel uterine arteries, severe pregnancy complications such as pregnancy loss, IUGR, and PE occur.

Trophoblast cells are often compared to invasive carcinoma cells since both can invade, migrate, and evade immune response [68]. Increasing evidence has revealed the key roles of lncRNAs in proliferation, survival, angiogenesis, migration, and cancer cell invasion [69]. In the last few years, functional studies on well-characterized choriocarcinoma cell lines representing villous and extravillous trophoblast phenotype (BeWo, JAR, and JEG-3), first-trimester trophoblast cell lines (HTR-8/SVneo and Swan-71), and primary trophoblast cell cultures reported some lncRNAs involved in regulatory mechanisms in trophoblasts activity, such as H19, HOTAIR, MALAT1, SPRY4-IT1, and MEG3 [70]. The potential mechanisms underlying the action of these and others lncRNAs in trophoblasts will be discussed in each pathology to elucidate the potential role of these molecules.

## 4. LncRNAs in Preeclampsia

Dysfunctional placentation is believed to be an underlying vital cause of PE. The placenta consists of the fetal portion, which predominantly involves trophoblastic cell proliferation, migration, invasion, and the maternal component, involving decidualization, crucial for the development of the placenta. From the fetal portion of the placenta, several lncRNAs regulate or influence the cell cycle, then affecting trophoblastic cell proliferation, invasion, migration, and apoptosis, leading to placental dysfunction and PE [71]. Numerous reports have described the aberrant expression of lncRNAs in plasma or placentae samples collected from women with PE (Figure 1). For instance, the lncRNA SH3PXD2A-AS1, which was found upregulated in term placentae from PE women, is reported to be involved in placenta development by recruiting the CCCTC-binding factor (CTCF) to the promoter regions of SH3PXD2A and CCR7, thus inhibiting the transcription of these two essential factors that are involved in invasion and migration of early trophoblast cells [70]. Another lncRNA described as upregulated in placentas from PE patients is the lncRNA H19 [72]. This molecule spans 2600-nt and predominantly resides in the cytoplasm with a minor fraction in the nucleus [73]. Moreover, lncRNA H19 is regulated by paternal imprinting (maternally expressed) in villous, interstitial trophoblast, cytotrophoblast, and syncytiotrophoblast [74]. Interestingly, overexpression of H19 in JEG-3 and HTR-8/SVneo cells reduced cell viability and increased invasion and autophagy accompanied by the activation of the PI3K/AKT/mTOR pathways [72]. Zuo et al. demonstrated that the lncRNA SPRY4 intronic transcript 1 (SPRY4-IT1) regulated trophoblast cell invasion and migration by affecting the epithelial–mesenchymal transition. Specifically, the authors demonstrated that SPRY4-IT1 binds directly to HuR and mediates the β-catenin expression associated with the epithelial–mesenchymal transition in HTR-8/SVneo cells [75]. A different study further demonstrated that the silencing of lncRNA SPRY4-IT1 in HTR-8/SVneo cells enhanced cell migration and proliferation but reduced the response of these cells to apoptosis. Moreover, the expression of this lncRNA was higher in severe preeclamptic placentae as compared with normal placentae, suggesting that SPRY4-IT1 might be associated with the pathogenesis of PE and might provide a new target for its early diagnosis and treatment [76].

Similarly, several other lncRNAs have been reported aberrantly overexpressed in PE placental tissues: The lncRNA MIR503 host gene (MIR503HG), lncRNA INHBA-AS1, and lncRNA uc003fir, which are involved in cell proliferation, invasion, and migration [77,78,79],

Among the lncRNAs that are downregulated in PE placentae MALAT1 [80], TUG1 [81], MEG3 [82], and HOXA11-AS [83] showed an increase in cell arrest and apoptosis and a decrease in cell proliferation and migration. RNA-seq analysis further indicated that HOXA11-AS silencing preferentially regulated genes associated with trophoblast migration and proliferation through association with repressive chromatin factors such as Ezh2 and Lsd1 or by acting as miRNA sponge [83]. Furthermore, gain-of-function experiments with TDGR1 and ZEB2-AS1, two lncRNAs related to developing different cancer tumors, have demonstrated an accelerated proliferation, migration, and invasion of trophoblast cells [84,85]. In addition, Yin et al. has recently demonstrated that the expression of the lncRNA-ATB was significantly lower in placenta tissues from preeclampsia patients, compared to controls [86]. The LINC00473 is another example of a lncRNAs downregulated in PE. This RNA regulates the invasion and migration of the trophoblast through Lsd1 and miR-15a-5p [87,88].

Impaired placentation due to deficient trophoblast invasion leads to an impaired uterine spiral artery remodeling and angiogenesis. Metastasis-associated lung adenocarcinoma transcript-1 (MALAT1) lncRNA knockdown in HTR-8/SVneo suppressed migration and invasion of these cells and inhibited tubule formation in endothelial cells when co-cultured with trophoblast cells. This impairment of angiogenesis through MALAT1 silencing was due to the downregulation of the angiogenic factor VEGF [89]. The suppressed migration, invasiveness, and proliferation observed when MALAT-1 is silenced are also observed in experiments using JEG-3 cells. Interestingly, this silencing induced cell cycle arrest at the G_0_/G_1_ phase resulting in enhanced JEG-3 cells apoptosis. This process was accompanied by elevated levels of the pro-apoptotic proteins, including cleaved caspase-3, cleaved caspase-9, and cleaved poly (ADP-ribose) polymerase-1 (PARP-1) [80]. A different study confirmed these findings and further demonstrated that MALAT1 promotes trophoblast migration and invasion through FOS-induced EMT, highlighting new roles for MALAT1 in spiral artery remodeling and its potential involvement in the pathogenesis of PE [90]. Lastly, Wu et al. have identified that MALAT1 regulates the miR-206/IGF-1 axis, thereby modulating trophoblast cells’ migration and invasion through the PI3K/Akt signal pathway [91].

From the maternal portion of the placenta, poor decidualization and spiral artery remodeling could result in placental ischemia and PE [92,93]. The lncRNA HK2P1 and its cognate gene HK2 were described as critical for glycolysis, angiogenesis, and decidualization, showing a decreased expression in the decidua of severe PE [94].

A summary of the expression of lncRNAs in PE placentae is shown in Table 1.

## 5. LncRNA Regulation in the Decidua and Peripheral Blood

Different studies have shown differential expression of lncRNAs in the decidua of patients with a history of PE. For example, Tong et al. studied the decidua basalis tissue of early-onset PE (<34 weeks of gestation at delivery) and late-onset PE (>34 weeks of gestation). They showed 32 differentially expressed lncRNAs, including the expression of H19, which was significantly downregulated in term placentae of early-onset severe PE patients [116]. Furthermore, H19 rs2107425 polymorphism was associated with a higher risk of PE; on the other hand, the placental promoter hypermethylation of the H19 gene was associated with a lower risk of PE [117].

An imbalance of the immune reaction in pregnancy, which predominantly involves T cells, is thought to play an essential role in the development of PE. Dendritic cells (DCs) are involved in the development of pregnancy immune tolerance. Lnc-DC is exclusively expressed in human DCs and can affect the differentiation from monocytes and influence T cell activation by activating the transcription factor STAT3 [118]. It has been shown that T regulatory cells and dendritic cells are significantly decreased in the peripheral blood of women with PE [119] but also found to be increased in the decidua of PE patients. Zhang et al. found the expression of lnc-DC and p-STAT 3 to be increased in the decidua of PE patients, suggesting over-maturation of decidual dendritic cells and a more pro-inflammatory response as a possible underlying etiopathology for PE [120].

Finally, the changes in the expression of lncRNAs in peripheral maternal blood during pregnancy have been proposed as possible biomarkers of PE. The expression level of NONHSAT116812 and NONHSAT145880 that was significantly lower and higher, respectively, in term placentae of PE women, strongly correlated with their expression on the plasma of the same women 48h before delivery [121].

## 6. LncRNA and Intrauterine Growth Restriction

The role of lncRNAs in IUGR have been less studied than in PE. However, like in PE, disordered placentation is believed to be the underlying pathophysiology of IUGR. Hence, it is essential to establish whether dysregulation of lncRNAs could also play a role in the physiopathology of IUGR.

Several lncRNAs have shown differential expression in placentae of IUGR vs. normally grown fetuses. Medina-Bastidas et al. [122] identified a decrease of 98 lncRNAs and an increase of 36 lncRNAs in IUGR-placentae compared to normal ones, evidencing the potentially critical role of the lncRNA as part of the pathophysiology of this disease. However, few of them have been thoroughly studied, which makes it difficult to understand their definitive role in the etiopathology of the disease. From the placental dysfunction perspective, the nuclear paraspeckle assembly transcript 1 (NEAT1) [123,124], abnormally upregulated in cancer, has shown increased expression in the placentas of fetal-growth-restricted fetuses. The increase in NEAT1 is thought to increase paraspeckles in villous trophoblasts, leading to increased retention of mRNAs in their nuclei. Another lncRNA, the MEG3 gene, suppresses cell growth and increases tumor suppressor p53. Placentae of fetuses with intrauterine growth restriction were found to have decreased MEG3 expression [125]. As villous trophoblasts are crucial for placental development, this could lead to placental dysfunction and fetal growth restriction. However, the exact causal mechanism is unknown, and this association was not consistent in other studies [124].

Associations between pancreatic islet cell dysfunction/insulin production and fetal growth restriction have been reported [126]. The taurine upregulated gene 1 (TUG1) is a lncRNA that is believed to participate in cell growth and islet cell dysfunction in a model of growth-restricted mice [127]. Moreover, silencing of TUG1 resulted in increased apoptosis and decreased insulin secretion in pancreatic β cells [128], thus possibly influencing fetal growth.

As in PE, epigenetics may play a significant role in the development of IUGR. Alteration of the H19 locus has been associated with fetal and placental growth pathology [129,130], and in murine models lacking H19, they show a significant fetal/placenta overgrowth [131]. H19 expression is regulated by the levels of methylation of CpG islands, and the methylation levels of the H19 gene increase during pregnancy [132]. Alteration in the methylation degree mechanism has been described in PE and IUGR [132]. Interestingly, H19 is a multifunctional lncRNA that exerts its functions by (i) miRNA production of miR-675, an evolutionarily conserved cell proliferation regulator and a placental growth inhibitor [130,131], (ii) miRNA sponge for let-7 miRNA [133], and (iii) recruiting methyl-CpG-binding domain protein 1 (MBD1), a DNA-methylation–dependent transcriptional repressor [134]. On the other hand, H19 repression affects trophoblast migration and invasion [73]. Gonzalez-Rodriguez et al. found that heritable growth restriction was associated with changes in H19 gene expression and were reversible with diet supplementation in the long term [73,135].

## 7. LncRNAs and Other Pregnancy-Related Disorders

Placenta previa increta/percreta (I/P) is a severe form of invasive placentation associated with massive peripartum hemorrhage. The pathogenesis of invasive placentation is multidimensional, involving decidual deficiency, endomyometrial damage, and excessively deep trophoblast invasion into the uterus [136]. Opposite to PE, which is associated with shallow trophoblast invasion, Tseng and colleagues reported a significant overexpression of MALAT-1 in I/P term placentae, compared to healthy pregnancy controls. Silencing of this lncRNA in three trophoblast-like choriocarcinoma cells (BeWo, JAR, and JEG-3) was sufficient to suppress the invasive ability of these cells, a process that was not correlated with abnormal MMP-2 and MMP-9 enzyme activities [136]. These results suggest that MALAT-1 might be involved in the regulation of trophoblast invasion during the development of advanced invasive placentation. The maternally expressed gene 3 (MEG3) is another lncRNA found to be downregulated in placental samples from PE patients compared to samples collected from normotensive patients, and abnormal levels of MEG3 were shown to result in cellular dysfunctions of HTR-8/SVneo and JEG-3 trophoblast cells [82]. Recently, Wang et al. have shown that ncRNA MEG3 is also involved in the epithelial–mesenchymal transition in HTR-8/SVneo cells through miR-210, affecting the activity of trophoblast migration and invasion [137].

Recurrent spontaneous abortion (RSA) is a common complication of human pregnancy defined by two or more abortions before the 20th gestational week. The incidence of RSA is approximately 1–5%, with an increasing tendency in recent years [138]. RSA occurrence is associated with trophoblast insufficiency and impaired remodeling of the spiral arteries into low-resistance blood vessels. Yang et al. has recently reported that the expression of the lncRNA-PVT1 is significantly reduced in tissues from RSA patients [138]. They also demonstrated that knockdown of lncRNA-PVT1 significantly reduced adhesion, invasion, autophagy, and mTOR expression in HTR-8/SVneo cells, which significantly increased apoptosis. This study further revealed a novel regulatory pathway in which Yin Yang 1 (YY1), a transcription factor involved in the regulation of trophoblast invasiveness at the maternal-fetal interface, can act directly on the lncRNA-PVT1 promoter to regulate its transcription, which further affects trophoblast invasion by regulating autophagy via the mTOR pathway [138].

## 8. LncRNA Transportation in EVs

Extracellular vesicles (EVs) are a heterogeneous class of small, phospholipid-coated vesicles released into the extracellular environment by most cell types; this class includes several subpopulations such as apoptotic bodies (500 nm to 2 µm diameter), microvesicles (100 to 1000 nm diameter), and exosomes (30 to 150 nm diameter) [139,140,141]. Both classes of EVs contain a complex cargo with different types of biomolecules such as proteins, lipids, DNA, and RNA, which can act as intercellular messengers and coordinate critical biological responses of the target cells [141,142,143]. Interestingly, the release and content of EVs are related to the cell type and differ in normal and pathological states [144,145,146,147,148]. Furthermore, different EVs have been identified, detected, and isolated from various body fluids like blood, saliva, milk, amniotic liquid, lymph, and lachrymal [141,149]. Therefore, EVs and their cargo can be used as pathology biomarkers if they can be detected effectively and differ between normal and diseased tissue.

EV-associated RNAs are one of the most studied areas in EV research. Several studies suggest that EV-associated RNAs such as miRNAs or lncRNAs have been shown to mediate intercellular communication to be functional in recipient cells [142,150,151,152]. Diverse groups have described the role of miRNAs associated with EVs, being the most well-studied RNA molecule in EVs [142,148,153,154,155]. On the other hand, little is known about the role of lncRNAs carried by EVs; however, these long RNAs are increasingly recognized as essential mediators of EVs biological effects [156,157,158,159]. For example, Chen and colleagues have demonstrated that exosomes derived from mesenchymal stem cells (MSCs) from bone marrow origin carry the lncRNA H19, which can be transferred to trophoblast cells and activate the protein kinase B (AKT) signaling pathway, thus increasing invasion and migration and inhibiting apoptosis of HTR-8/SVneo trophoblast cells. These results suggest that MSC-derived exosomes overexpressing H19 may be a novel direction for therapeutic strategies against PE [157].

The placenta increases and releases a wide variety of EVs and molecules, supporting the maternal physiology to adjust to fetal requirements during gestation. While identifying EVs-associated miRNAs has started to be used as new pregnancy disease biomarkers, the functional role of EVs-associated lncRNAs remains unknown, mainly in these pathologies.

## 9. Conclusions

Pregnancy complications such as preeclampsia and intrauterine growth restriction contribute highly to maternal and fetal morbidity and mortality worldwide. Unfortunately, such complications often remain undetected until the third trimester. Understanding the origins and causes of these disorders and implementing effective screening will significantly improve the outcomes of those pregnancies. In recent years, lncRNAs have gained importance as regulators of gene expression in several biological processes in humans. A growing body of evidence has reported the crucial involvement of lncRNAs in trophoblast cells proliferation, invasion, migration, and apoptosis, which has inevitably linked lncRNAs to the onset of placental-related disorders. The identification of lncRNAs related to normal and abnormal placental development can help understand the pathogenesis of PE/IUGR complications and help develop new prediction and therapeutic strategies.

## Figures and Tables

**Table 1:** Summary of lncRNAs associated with PE.

lncRNA	Expression in PE	Cells or Tissue Analyzed	Intersection Molecules and Pathways	Cellular Processes (Cell Line)	References
CAAT1	Up	Term placentae, PE	Reduces the expression of E2F1, cyclin D	Decreases proliferation (JEG-3)	[95]
DLX6-AS1	Up	Term placentae, PE	Enhances ERP44 expression by sponging miR-149–5p	Inhibits proliferation, invasion, angiogenesis; promotes cell apoptosis (HTR-8/SVneo, JEG-3)	[96]
GAS5	Up	Term placentae, PE (C-section)	Regulates miR-21; activates PI3K/AKT signaling pathway	Inhibits proliferation, migration, invasion (HTR-8/SVneo JEG-3)	[97]
H19	Up	Term placentae, PE	Activates PI3K/AKT/mTOR pathways	Promotes invasion and autophagy; reduces cell viability (HTR-8/SVneo, JEG-3)	[72]
HEIPP	Up	Term placentae, PE (C-section)	Unknown	Silencing HEIPP promotes invasion (HTR-8/SVneo)	[98]
HOTAIR	Up	Term placentae, PE	Unknown	Regulation of proliferation, invasion, and apoptosis (HTR-8/SVneo)	[99]
INHBA-AS1	Up	Term placentae, ePE (C-section)	Directly targets CENPB	Inhibits proliferation, migration, and invasion; promotes apoptosis (HTR-8/SVneo)	[78]
Linc00261	Up	Term placentae, severe PE (C-section)	Targets the mir-558/TIMP4 axis	Suppressed cell invasion and migration, induced cell apoptosis (HTR-8/SVneo cells)	[100]
MIR503HG	Up	Term placentae, severe PE (C-section)	Inhibits NF-κb pathway; decreases protein levels of MMP2, MMP9, and snail; increases protein level of E-cadherin	Suppresses proliferation, invasion, migration; promotes apoptosis (HTR-8/SVneo, JEG-3)	[77]
NR_002794	Up	Term placentae, PE	Unknown	Inhibits proliferation, migration; promotes apoptosis (Swan-71)	[101]
RPAIN	Up	Term placentae, epe (c-section)	Reduces the expression of c1q	Inhibits proliferation and invasion; promotes apoptosis (HTR-8/SVneo)	[102]
SH3PXD2A-AS1	Up	Term placentae, PE	Inhibits SH3PXD2A and CCR7 transcription	Inhibits invasion, migration, proliferation; promotes cell death (HTR8/SVneo)	[70]
SPRY4-IT1	Up	Term placentae, PE (C-section)	Binds directly to Hur and mediates EMT	Inhibits invasion and migration (HTR-8/SVneo)	[75]
SPRY4-IT1	Up	Term placentae, severe PE (C-section)	Unknown	Inhibits proliferation, migration, angiogenesis; enhances apoptosis (HTR-8/SVneo)	[76]
uc003fir	Up	Term placentae, PE	Promotes CCL5 mRNA expression	Promotes proliferation, migration, invasion (HTR-8/SVneo)	[79]
UCA1	Up	Term placentae, PE (C-section)	Directly targets JAK2	Silencing of UCA1 promotes proliferation, invasion; suppresses apoptosis (HTR-8/SVneo, JAR)	[103]
Linc00473	Down	Term placentae, PE (C-section)	Inhibits expression of TFPI2 through binding to LSD1	Inhibits cell proliferation, migration, invasion, angiogenesis; promotes apoptosis (HTR-8/SVneo)	[87]
AGAP2-AS1	Down	Term placentae, PE	Direct target of FOXP1	Inhibits proliferation, migration, invasion; promotes apoptosis (HTR-8/SVneo)	[104]
AK002210	Down	Term placentae and blood, PE	Mir-590-3p directly targets AK002210	Inhibits proliferation, migration, invasion (HTR-8/SVneo)	[105]
CRNDE	Down	Term placentae, PE (C-section)	Negatively regulates mir-1277	Suppresses proliferation, invasion, migration and EMT formation (HTR-8/SVneo)	[106]
FAM99A	Down	Term placentae, PE (C-section)	Increases expression of cleaved caspase-3, -9 and Bax; inhibits Wnt/β-catenin signaling	Suppresses migration, invasion; increases apoptosis (HTR-8/SVneo)	[107]
FOXD2-AS1	Down	Plasma, PE	Suppresses expression of MMP2 and MMP9	Inhibits proliferation, invasion, migration (HTR-8/SVneo)	[108]
GASAL1	Down	Term placentae, PE (C-section)	Directly binds to SRSF1 protein	Inhibits proliferation, invasion; increases apoptosis rate (HTR-8/SVneo, JAR)	[109]
HIF1A-AS2	Down	Term placentae, PE (C-section)	Recruits LSD1 and epigenetically represses PHLDA1	Inhibits proliferation, migration, invasion; increases apoptosis (HTR-8/SVneo, JAR)	[110]
HOXA11-AS	Down	Term placentae, PE	Recruits Ezh2 and Lsd1 proteins to silence RND3 mRNA	Suppresses proliferation, migration (HTR-8/SVneo, JEG-3, JAR)	[83]
LINC00511	Down	Term placentae, PE (C-section)	Direct target of AP2γ	Insufficient proliferation, migration, invasion (HTR-8/SVneo)	[111]
MALAT1	Down	Term placentae, PE	Unknown	Suppresses proliferation, migration, invasion; induces apoptosis (JEG-3)	[80]
MALAT1	Down	Term placentae, PE	Unknown	Suppresses proliferation, migration, invasion, angiogenesis; induces apoptosis (HTR-8/SVneo)	[89]
MALAT1	Down	Term placentae, ePE (C-section)	Targets FOS	Inhibits migration and invasion (HTR-8/SVneo, JAR)	[90]
MALAT1	Down	Term placentae, PE (C-section)	Regulates IGF-1/PI3K/Akt signaling via directly binding to mir-206	Restrains migration and invasion (HTR-8/SVneo, JEG-3)	[91]
MEG3	Down	Term placentae, PE (C-section)	NF-kb, Caspase-3, and Bax increased following MEG3 knockdown	Decreases migration; increases apoptosis (HTR-8/SVneo, JEG-3)	[82]
MVIH	Down	Term placentae, PE	Unknown	Inhibits proliferation, migration, and invasion (HTR-8/SVneo, JEG-3)	[112]
SNHG12	Down	Term placentae, PE	Promotes expression of MMP-2 and MMP-9, β-catenin	Inhibits proliferation, migration, invasion (HTR-8/SVneo)	[113]
SNHG14	Down	Blood, PE	Negatively regulates mir-330-5p	Suppresses proliferation, migration, invasion, EMT (HTR-8/SVneo)	[114]
TDRG1	Down	Term placentae, PE (C-section)	Targets and suppresses mir-214-5p	Inhibits proliferation, migration, invasion (JEG-3)	[84]
TUG1	Down	Term placentae, PE (C-section)	Regulates MCL1, VEGFA, and MMP2 through targeting mir-29b	Inhibits proliferation, invasion, angiogenesis; promotes apoptosis (HTR-8/SVneo, BeWo)	[115]
TUG1	Down	Term placentae, PE (C-section)	Inhibits RND3 through binding to EZH2	Inhibits proliferation, apoptosis, migration, angiogenesis; increases apoptosis (HTR-8/SVneo, JEG-3)	[81]
ZEB2-AS1	Down	Term placentae, severe PE	Affects mir-149/PGF axis	Suppresses proliferation, migration, invasion (HTR-8/SVneo)	[85]

Table Abbreviations: lncRNA, long noncoding RNA; Up, upregulated; Down, downregulated; PE, Preeclampsia; ePE, early-onset Preeclampsia EMT, epithelial-to-mesenchymal transition; MMP, matrix metalloproteinase; C-section, Caesarean section.

## Data Availability

Not applicable.
